# T83. PROCESSING SPEED PERFORMANCE AND FUNCTIONING IN YOUNG ADOLESCENTS EXPERIENCING AUDITORY HALLUCINATIONS

**DOI:** 10.1093/schbul/sby016.359

**Published:** 2018-04-01

**Authors:** Joanne Kenney, Ian Kelleher, Colm Healy, Niamh Dooley, Eleanor Carey, Amy Adair, Donal Campbell, Helen Coughlan, Erik O’ Hanlon, Mary Cannon

**Affiliations:** Royal College of Surgeons in Irel

## Abstract

**Background:**

Neurocognitive impairments are a prevalent aspect of psychosis which, on average, begin in early adolescents, with particular impairment apparent in speed of processing and nonverbal working memory in early stages (Kelleher et al., 2012). It is important to understand the impact of cognitive impairment on functional ability, particularly in early stages of illness which may assist in the development of targeted therapeutic strategies.

**Methods:**

A population sample of 212 school going adolescents aged 11–13 years partook in the study, which included community-based adolescents who report experiencing psychotic symptoms but who were not clinically diagnosed. Psychotic symptoms were assessed using the psychosis section of the Schedule for Affective Disorders and Schizophrenia. Six cognitive domains were assessed using the MATRICS consensus cognitive battery. Functioning was assessed using the Children’s Global Assessment Scale. Six separate linear regression analyses were performed to test if each cognitive domain of the MATRICS battery predicted functioning.

**Results:**

In the entire sample (including those who experienced psychotic experiences and those who did not) (n=211), speed of processing significantly explained 8% of the variance in functioning (F(1, 76) = 6.61, p = .0012, R-squared = 0.08.), (Beta = 0.39, p = 0.012). When the sample was subdivided into those who ever experienced auditory hallucinations (AH) (n=62) versus those that did not (n=149), speed of processing significantly predicted 18% of the variance in functioning in the group experiencing AHs (F(2, 33) = 3.82, p =0.032, R-squared = 0.18), (Beta = 0.43, p = 0.06). However, no effect was found in the group without AVs (F(1,40) = 1.19, p =0.28). No other cognitive domain predicted functioning.

**Discussion:**

Speed of processing appears to be a core cognitive deficit in psychosis which impacts on functioning in young adolescents particularly in those experiencing psychotic symptoms such as auditory hallucinations, however the variance predicted by processing speed is relatively low. This research highlights the potential of speed of processing as a possible viable target for early intervention in psychotic disorders.

